# College and Hospital Notes

**Published:** 1896-08

**Authors:** 


### College and Hospital Notes.

The fifteenth annual announcement of the New York Post-Graduate Medical School and Hospital has just been issued. Five hundred and forty-two physicians from all over this continent have attended the courses at the institution during the past year. More than 1,000 operations were performed in the hospital, which is one of the largest in the city, containing special wards for babies and children, while nearly 20,000 patients were treated in the out-door department. Recent discoveries have revolutionised medical and surgical methods and a man whose medical education ended fifteen years ago is not a physician and surgeon within the present meaning of the term. Post-graduate medical instruction is for the purpose of furnishing to these graduates in medicine a means of refreshing their knowledge. It supplies them with the opportunity of coming in direct contact with disease by means of special courses in all the departments of medicine.

---

The Buffalo State Hospital has in contemplation enlarged accommodations in the nature of new buildings to be called the Infirmary of the Buffalo State Hospital. It will be for the accommodation of the insane sick of western New York and will contain 260 beds, capable of being increased to 300 beds in an emergency. There will be one large central administration building, in which an assistant physician will reside, and in which there will be administration offices and a chemical laboratory. On the second floor there will be a large clinic room, in which distinguished alienists will be invited to lecture. The whole will be under the care of Dr. A. W. Hurd, superintendent of the State Hospital, whose well-known administrative ability and skill as an alienist is a guarantee of continued successful management of the hospital as enlarged.

---

The City Hospital for Women is an institution lately established at 859 Humboldt parkway by Dr. Charles E. Congdon. The location is one of the most healthful in the city of Buffalo, and there is every reason why the hospital should make a good record. It will train its own nurses and avail itself of all other methods of modern hospital management. Attention is invited to the card of the hospital in our advertising pages.

